# What Is COVID 19 Teaching Us about Pulmonary Ultrasound?

**DOI:** 10.3390/diagnostics12040838

**Published:** 2022-03-29

**Authors:** Gino Soldati, Marcello Demi

**Affiliations:** 1 Ippocrate Medical Center, 55032 Lucca, Italy; soldatigino@yahoo.it; 2 Department of Bioengineering, Fondazione Toscana Gabriele Monasterio, 56126 Pisa, Italy

**Keywords:** COVID-19, clinical review, lung ultrasound imaging

## Abstract

In lung ultrasound (LUS), the interactions between the acoustic pulse and the lung surface (including the pleura and a small subpleural layer of tissue) are crucial. Variations of the peripheral lung density and the subpleural alveolar shape and its configuration are typically connected to the presence of ultrasound artifacts and consolidations. COVID-19 pneumonia can give rise to a variety of pathological pulmonary changes ranging from mild diffuse alveolar damage (DAD) to severe acute respiratory distress syndrome (ARDS), characterized by peripheral bilateral patchy lung involvement. These findings are well described in CT imaging and in anatomopathological cases. Ultrasound artifacts and consolidations are therefore expected signs in COVID-19 pneumonia because edema, DAD, lung hemorrhage, interstitial thickening, hyaline membranes, and infiltrative lung diseases when they arise in a subpleural position, generate ultrasound findings. This review analyzes the structure of the ultrasound images in the normal and pathological lung given our current knowledge, and the role of LUS in the diagnosis and monitoring of patients with COVID-19 lung involvement.

## 1. Introduction

Chest ultrasonography is gaining ever more consideration among physicians as a useful diagnostic tool. In recent years, cardiologists, intensivists, and many pulmonologists have explored the diagnostic capability of ultrasound in chest diseases.

Novel aspects in clinical methodology regarding how to approach respiratory patients with the help of ultrasound have already been discussed in literature [1,2].

Moreover, recent studies have furthered our knowledge of the physical mechanisms underlying the formation of the images in lung ultrasound (LUS) [3,4,5,6,7,8,9,10]. 

The recent COVID-19 pandemic has contributed to expanding the indications for the use of ultrasound to assess and monitor viral lung lesions. However, problems have also emerged relating to the poor specificity of the ultrasound findings reported in the pulmonary involvement of COVID-19 [11] and the need for better knowledge of the ultrasound signs connected to the interstitial diseases (which are notoriously represented by artifacts) strongly emerged.

Similarly to what has been already done to distinguish between primary pulmonary and secondary cardiogenic lung pathology [12], a valuable strategy is that of integrating the morphological data related to ultrasound and data derived from both the clinical examination and the epidemiological context.

Knowledge of the relationship between artefactual images and the superficial histology of the lung, in terms of airspace distribution [5], represents a field of extreme interest since this can improve the low specificity of ultrasound signs of sonographic interstitial syndrome (SIS).

Ultimately, the ultrasound semeiotics of the lung are based on artefactual findings and anatomical images (consolidations) [13].

Lung artifacts carry important information on the subpleural density and subpleural structural disorder [5,14,15]. The more the lung is similar to air, the more evident the replicas of the pleural line (generically called A-Lines) and the replica and mirror effects of the thoracic wall structures will be. Conversely, the denser the pulmonary subpleural layer is, the more vertical artifacts (known as B-Lines) and white lung will be visualized. 

B-lines represent a large variety of vertical artifacts that identify a wide range of superficial lung diseases that do not consolidate the organ. The pathology is represented by edema (cardiogenic and non), non-consolidating pneumonia, and interstitial lung diseases and by all those conditions that are able to modify the subpleural ratio between full and empty, in favor of the former. Lung consolidations appear when the lung is severely air-deprived [13].

Based on current knowledge, in this review we will synthetize the physical basis of the artifacts and consolidations that are observed in LUS images and the relationships of these findings with the explored histopathology in COVID-19 pulmonary damage. 

COVID-19 pneumonia has a macro/micromorphology and histopathological pattern of which we have sufficient knowledge through CT images and postmortem examinations, and its prevailing anatomical picture is an inhomogeneous increase of subpleural density (due to interstitial thickening and consolidation). Consequently, the experience acquired in the laboratory along with the clinical use of LUS can be used for the LUS interpretation of a COVID-19 pulmonary injury.

### 1.1. Historical Perspective of Ultrasound (US) Vertical Artifacts

While the meaning and genesis of A-Lines and consolidations are well known, in this section special emphasis will be given to the significance of vertical lung artifacts, generally known as B-Lines.

References to vertical artifacts in ultrasound imaging date back to the 1980s. “Comet-tail artifacts” were described in abdominal and thoracic ultrasound images even though a clear explanation regarding their origin was not provided [16,17]. 

In 1985 Avruch explained the origin of vertical artifacts in experimental models as a “resonance” phenomenon occurring when the ultrasound enters a fluid film surrounded by a tetrahedral disposition of bubbles. These artifacts were named “ring-down artifacts” [18]. 

In their seminal work, Lichtenstein and co-workers described pulmonary “comet-tail artifacts” related to pulmonary edema [19,20]. By comparing ultrasound images with chest CT scans at the same level, an association between these artifacts (subsequently called B-Lines) and thickened interlobular septa was observed. With these observations as a starting point an association between the “comet-tail artifacts” or B-Lines and increased pulmonary extravascular water was speculated. Many studies observed a correlation between the number of vertical artifacts and the severity or the evolution of the cardiogenic pulmonary edema [21]. 

In many cardiological and critical care settings, the presence of “comet-tail artifacts” was considered synonymous with the presence of pulmonary extravascular water, even if the evidence showed that these artifacts were present in many pathological conditions of the pulmonary interstitium [22].

More recently, the hypothesis that the US vertical artifacts do not represent discrete anatomical structures of the lung, but are the expression of the ultrasound interaction on a lung surface (the immediately subpleural tissue), which is denser than normal but not yet consolidated [3,7], has been proposed.

In other words, several anatomic structures may be responsible for the genesis of the artifacts. The requirement for this is essentially the existence of acoustically permissive structures (acoustic channels and traps) surrounded by aerated spaces and linked to the pleura. In this sense, not only may the thickened interlobular septa be responsible for the vertical artifacts [23,24], but micronodules, groups of collapsed alveoli, neo production of collagen, and so on, could also be responsible.

A clear demonstration of this is the possibility of reproducing similar artifacts in non-biological materials [10,24] and in healthy lungs when deflated to a critical, non-physiological level of density [14].

### 1.2. An Introduction to the Clinical Use of Artifacts

Traditionally, the most important artefactual patterns in LUS are A-lines, B-lines, and white lung [12,13].

**Figure 1:**
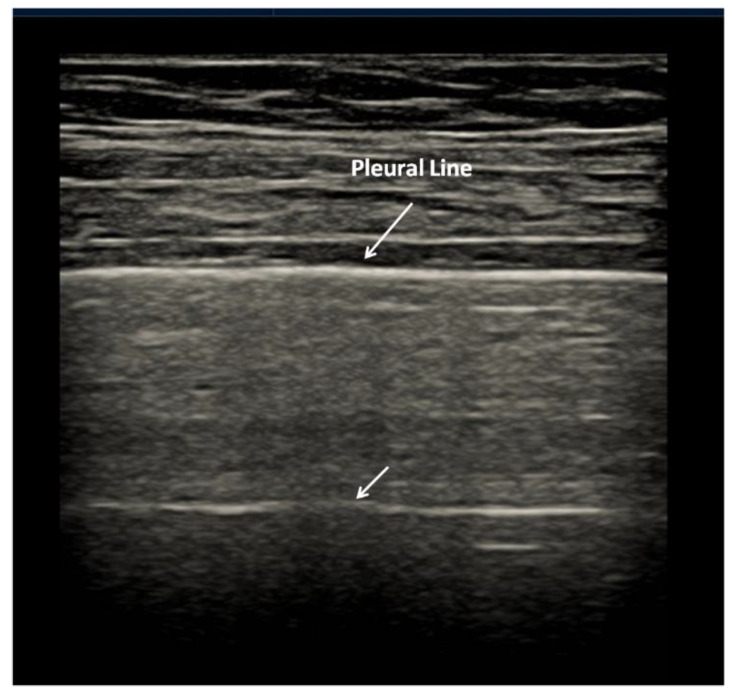
Normal lung. Pleural line is regular. The first artefactual replica of the pleural line is clearly seen (deeper arrow). Between the pleural line and the first A-line, a blurred superposition of the parietal acoustic discontinuities appears due to the mirror and replica effects caused by the strong reflection of the pleural line. Linear probe, 8 MHz.

A-lines are horizontal artifacts related to a normal pleural plane. A-lines are a replica of the pleural line and a blurred superposition of the parietal acoustic discontinuities appears between the pleura line and the first A-line (as well as between every pair of subsequent A-lines) due to the mirror and replica effects caused by the strong reflection of the pleural line [7], (Figure 1). This pattern represents how ultrasound scanners visualize the echo signals that are bouncing between the probe, the chest wall planes, and the lung surface.

The expression of the A-lines is however closely linked to the acoustic energy incident on the pleural plane and to its reflectivity, and introduces further useful elements for better ultrasound semeiotics of the lung, as it is possible that in many lung pathologies the reflectivity of the pleural line may be impaired (see later) [4,7,10].

As already mentioned, the so-called B-Lines are linked to the existence of acoustic traps distributed along the pleural surface, which are capable of capturing the acoustic energy among the aerated spaces and returning it as a prolonged signal over time. According to this view, acoustic energy can be partially trapped and subsequently re-radiated towards the probe after multiple reflections between the separated aerated spaces, giving rise to vertical artifacts [4,7,9,10].

**Figure 2:**
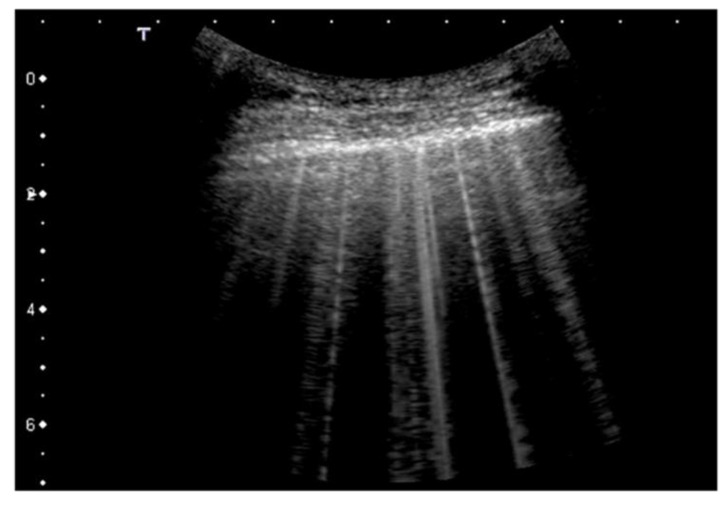
B-lines with variable appearance (cardiogenic pulmonary edema). B-lines are qualitatively characterized by their brightness, the full screen extension, the pleural origin, and the presence or absence of internal modulation. Convex probe, 6 MHz.

**Figure 3:**
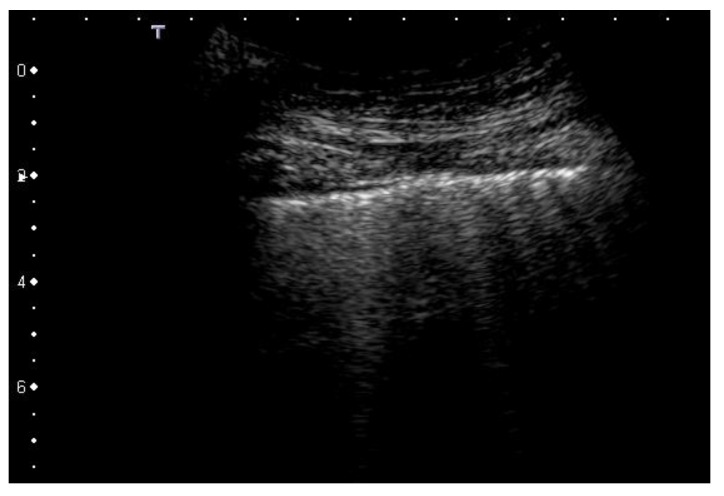
Vertical artifacts from a patient with scleroderma and pulmonary fibrosis. They show variable brightness, width, and length. Convex probe, 3 MHz.

Some recent studies have validated this hypothesis and introduced further elements for a more accurate definition of the relationships between the morphology of the artifacts and the distribution of the air spaces within the subpleural interstitium. In essence, this links the distribution and visual appearance of the different B-lines to the subpleural histology [5] (Figure 2 and Figure 3).

In our opinion, therefore, considerable importance is assumed by the morphology of the artifacts as it is shown that the configuration of the acoustic traps determines the appearance of the individual artifacts. A large list of phenomena and configurations acting as active acoustic channels along the pleural surface may play a crucial role in producing many different types of artifacts in many diffuse and localized superficial interstitial diseases, which depend on different re-arrangements of the air spaces. Therefore, every pair of acoustic channel and acoustic trap has its own spectral signature, which is related to the shape, size, and nature of the medium that constitutes the channel and the trap [6,7,10]. In practical terms, a visual inspection can show different vertical artifacts with respect to their pleural origin. Every vertical artifact has its own structure [5,20]. It can show a sequence of alternating white and black/gray horizontal bands (Figure 3) or a constant gray level, or it can appear more or less confused as has been illustrated by mathematical and physical models [7,9]. The width of the vertical artifacts is variable and this can even change from the start to the end point. 

The imaging parameters play a fundamental role in the formation of the artifacts, and the visibility of a vertical artifact depends on multiple non-orthogonal factors. Therefore, given the intrinsic variability of the artifacts as a function of multiple factors, making an objective diagnosis on the basis of the artefactual information is a difficult task when using the usual ultrasound devices [6].

Differences in the appearance of vertical artifacts between hydrostatic (cardiogenic) lung edema and adult respiratory distress syndrome (ARDS) were described some years ago [12] (Table 1). In light of recent evidence [25], the differences can be determined by the distribution and morphology of the acoustic traps generated in the two pathologies. A review on the ultrasound differential diagnosis of pulmonary and cardiac interstitial pathology reiterated some of these important concepts in light of current knowledge regarding the response of the pleural plane to ultrasound waves [26]. 

In some cases of particular subpleural echogenicity, the term “vertical artifacts” or B-Lines is not appropriate, even if the echogenic aspect may recall the coalescence of several vertical artifacts.

White lung [3,7,12] is a focal or multifocal LUS artifact, characterized by an undifferentiated echogenic background, with the absence of A-lines, and without clear evidence of vertical artifacts. This pattern suggests the presence of a relatively random scatterer distribution (many small airspaces close to each other which contribute to the formation of many small acoustic traps) which gives rise to a complex multiple scattering phenomenon. It appears to correlate with CT ground-glass attenuation. 

In conclusion, from a clinical point of view, lung pathological artifacts indicate a physical state of the subpleural lung that is denser, but which has not yet consolidated, caused by: (1) interstitial pathology enlarging the interstitial tissue but sparing residual peripheral air spaces; (2) pathological deflations of a normal healthy lung or pathological subversion of peripheral air spaces; or (3) mixed situations. In this way, the anatomic term “interstitial syndrome”, used to describe the presence of vertical artifacts could be changed to “hyperdense not-consolidated subpleural lung”. The spatial distribution and the variations in the morphology of the vertical artifacts can provide information on the structure of the subpleural lung at infra-millimeter dimensional levels. In the next sections the generic term of sonographic interstitial syndrome (SIS) is still used for historical reasons.

## 2. Clinical Interpretation of SIS

SIS is a non-specific echographic pattern. As it appears in various pathologies of the lung, its interpretation must include further information relating to its appearance and clinical context. Similarly, ultrasound COVID-19 findings, being also based on the presence of SIS, are not specific in themselves. To increase the specificity of ultrasound, when approaching SIS, it is important to focus on four steps in every situation:

1. Characteristics of the pleural line;
2. Characteristics of the artifacts;
3. Extension and distribution of SIS;
4. Relationships with clinical data and integrated multi-district sonography.

### 2.1. Characteristics of the Pleural Line

Once an US pulse reaches the visceral pleural surface it is near-totally reflected by the non-diseased lung because the size of the intra and interalveolar septa are relatively thin (with respect to the wave length of the carrier frequency). In these cases, the US pulse meets a sort of air wall, it is near-totally reflected towards the probe and the outer lung surface is represented as a thick white line where the thickness of this line is related to the length of the US pulse. It is necessary, here, to highlight that the perceived thickness of the pleural line is, in theory, equal to the length of the US pulse. However, this is true only if the direction of the wave propagation is orthogonal to the pleural plane. When the insonation is not orthogonal, and, even more importantly, when the visceral pleura is not “healthy” (in the presence of slightly thickened interstitial spaces, for example), it can appear blurred and apparently thickened.

In cases where the pleura is not a good acoustic reflector, even in the absence of vertical artifacts, the signs to be taken into consideration are the blurred and thickened appearance of the pleural line and, as a consequence, the less evident replica and mirror effects of the parietal structures below it.

The above is in agreement with known clinical experience. In ACPE, the pleural line is regular, smooth, linear, and with normal sliding. Generally, in primitive pulmonary interstitial diseases, the pleura contributes to the generation of artifacts, and is stably irregular, cobbled or even finely interrupted, especially in the basal regions [26,27,28]. Typical signs of ARDS are spared areas, and a normal or poorly altered pleural line with normal sliding next to areas with irregular pleura, which shows reduced or absent movements [29,30,31]. The pleural line is slight and focally irregular in score 1 (see Section 4) COVID-19, and becomes progressively more irregular as the disease progresses. 

### 2.2. Artifacts’ Characteristics

Different vertical artifacts are observed in acute cardiogenic pulmonary edema (ACPE), and acute respiratory distress syndrome (ARDS) or pulmonary fibrosis [3,29] (see Figure 3 and Table 1). 

In the case of ACPE (especially early ACPE), the lung architecture remains unchanged and shows only septal enlargement by transudate. The artifacts in early pulmonary edema are B-lines with their characteristic pleural point-like origin and brightness. In contrast, in pulmonary fibrosis, vertical artifacts are variable in morphology and distribution, and are often distinguishable by their low level of brightness and rapid attenuation.

In ARDS, the artefactual pattern is pneumogenic, inhomogeneous, and typically gravitational, with the most aerated lung in an elevated position and the denser or consolidated lung in the sloping position [32].

In COVID-19, a distribution of artifacts similar to that seen in early ARDS is present, but many single B-lines are brighter, and patchy columnar areas of white lung can be seen.

### 2.3. Extension and Distribution

SIS can be either focal, multifocal, or diffuse [27]. Mono- or oligofocal SIS is often seen around monolateral pulmonary consolidation, representing a denser but not consolidated tissue. This finding is suggestive of bacterial pneumonia.

When SIS is diffuse and bilateral, it is indicative of a diffuse pulmonary pathology. It can be either homogeneous or inhomogeneous. Homogeneous SIS can show a gravitational distribution, without spared areas. This could be indicative of cardiogenic pulmonary edema [12]. When SIS is bilateral and inhomogeneous, with spared areas, it could be indicative of non-cardiogenic pathology [22,26]. SIS in COVID-19 patients is typically pneumogenic and appears inhomogeneous and patchy, and is more prevalent in the basal portions of the lungs.

### 2.4. Relationships with Clinical Data and Integrated Multi-District Sonography

Given the non-specificity of vertical artifacts, it is only possible to suspect that one condition is more probable than another through an inferential abductive process of reasoning, which allows clinicians to make a more accurate diagnosis [1].

Knowledge of the clinical history and the preclinical probability of disease is useful and becomes crucial in the event of a COVID-19 epidemic. In the case of suspected cardiogenic SIS, echocardiography can add much information (cardiac signs of diastolic/systolic heart failure) [29]. Inferior caval vein dynamics give a rough preload estimate. A multi-district approach is useful when many diseases co-occur to establish a clinical picture or when cardiac or renal complications occur in COVID-19 patients. 

In the case of diffuse pneumogenic SIS associated with fibrotic interstitial lung diseases (ILDs), a typical appearance and distribution of artifacts with a congruent clinical picture (non-epidemic, subacute, or chronic onset) can aid in the diagnosis. Creating an acoustic pulmonary map is useful for narrowing down the diagnostic options, especially in diffuse ILDs [31].

## 3. Clinical Basis of COVID-19 Lung Ultrasound Imaging

Coronavirus disease 2019 (COVID-19) appeared in Wuhan, China, in December 2019, and rapidly increased to a pandemic level around the world. Its etiological agent is a novel coronavirus named severe acute respiratory syndrome coronavirus 2 (SARS-CoV-2) [33]. In the previous two decades, coronaviruses have caused other epidemic diseases—SARS-CoV-1 and Middle East respiratory syndrome (MERS-CoV). In SARS-CoV-1 coronavirus pneumonia, peripheral lung involvement was common but unifocal involvement was more common than multifocal or bilateral involvement. On CT images, GGOs with consolidations were the main findings, and reticulation was noted after the second week [34]. On CT images, MERS-CoV pneumonia showed subpleural and basilar airspace lesions, with extensive subpleural GGO and consolidation. Studies concerning the use of US in SARS-CoV-1 and in MERS-CoV are lacking. However, on the basis of the CT findings, we can suppose that the ultrasound signs in these pathologies would have been similar to those in patients with COVID-19. COVID-19, SARS-CoV-1, and MERS-CoV cause lung damage and, in their final stages, also multiorgan failure [35].

In general, in COVID-19 the most serious initial symptoms are related to pneumonia, while the evolution towards respiratory failure is similar to ARDS [36]. In light of our current knowledge regarding COVID-19, this picture may be too simplistic.

Many viruses cause pneumonia. Histopathology of viral pneumonia varies, and is related to the pathogenesis of pulmonary infection. Consequently, a computed tomographic (CT) pattern of viral pneumonia reports, at best, the fine pathology at the lobular level. Generally, interstitial viral pneumonitis shows a thickened interstitium with lymphocytic infiltration, and viral particles can be seen in both the bronchial and alveolar epithelium. Hyperplasia and desquamation of the alveolar lining cells and hemorrhage are the result of the harmful action of some viruses [37]. Histopathological findings in cases of SARS-CoV-1 and influenza infection (H1N1, H5N1) are characterized by diffuse alveolar damage (DAD), hemorrhage, edema, and hyaline membrane [31].

The histopathological picture of initial COVID-19 involvement is characterized by patchy DAD, interstitial thickness, and pneumocyte hyperplasia. Late stages show alveolar congestion, desquamation, organizing pneumonia, and hemorrhage [38,39,40]. More recent papers pay attention to the thickening of alveolar capillaries surrounded by edema, intraluminal fibrin thrombi, and CD61+ megakaryocytes in association with platelets [41]. Despite a direct viral infection of the endothelial cells being reported, other mechanisms of vascular involvement have been proposed. Magro et al. [42] examined lung tissue from five COVID-19 patients with respiratory failure. Histopathological patterns were characterized by significant capillary fibrin deposition. Vascular deposits of terminal complement components (C5-b9, C4d, MASP2) were noted, suggesting a systemic activation of a lectin-based complement pathway. Therefore, a complement-mediated coagulative dysregulation is possible. The proportion of COVID-19 patients with abnormal initial radiographic findings is low (50% or less). In these patients, chest computed tomography (CT) has a high sensitivity (97%) but a lower specificity (56%) for lung involvement, showing subpleural patchy ground-glass opacities (GGO), reticular and crazy paving patterns, and finally consolidations. A study demonstrated that lung involvement gradually increased to consolidation up to two weeks after the onset of the disease (72%, half of these with subsegmental appearance). In general, consolidations are considered an indication of disease progression [43,44].

When interpreting the lung ultrasound findings related to COVID-19, both the structural variations of the superficial lung tissue and CT findings are important. The former defines the appearance of the ultrasound images while the latter their sonographic visibility (only superficial alterations are visible with LUS).

In CT, GGO are present in 100% of cases, appearing in peripheral locations in 89% of cases. While 93% of patients have multilobar and posterior lung involvement, 91% of patients have bilateral findings [43]. These characteristics allow us to indicate ultrasound as a diagnostic tool in COVID-19 lung involvement. In accordance with the physical basis of the formation of ultrasound images, reticular and ground-glass opacities appear as artifacts, while the consolidative ones are anatomical tissue images [13,45].

Ultrasound reproduces superficial consolidations in explorable thoracic regions with excellent accuracy, including the presence of air and fluid bronchograms. Consolidations, that are evident in CT, appear on ultrasound if they emerge from the pleura. CT superficial interstitial thickenings appear on ultrasound as small consolidations or as vertical artifacts with variable lengths and modulations in relation to their shape and size. Ground-glass CT typically appears as white lung [3,4,13].

## 4. SIS in COVID-19

SIS is an expected finding in COVID-19 because edema, DAD, lung hemorrhage, interstitial thickening, hyaline membranes, and non-consolidative infiltrative lung diseases (if abutting the pleura) generate artefactual signs in LUS. In physical terms, the common denominator of these conditions is an increase in the density of the involved lung areas compared to the healthy lung [15]. The topographical distribution of COVID-19 findings, as visible in CT, justifies their appearance and the typically bilateral, multilobar, and patchy pattern [1]. The most affected lung areas are the posteroinferior (93.8%) followed by the lateral (88.7%) [46]. 

Many studies have addressed lung ultrasound findings in COVID-19 patients. Most of these adapted past experiences in other fields (intensive-care medicine and ARDS-CoV-1) to COVID-19 cases. In clinical practice, there are various ways to assess the extent of pulmonary involvement in COVID-19. In general, the larger the number of the explored areas, the greater the likelihood of a significant picture of overall lung involvement. 

A total of 12 areas over the chest, namely the anterosuperior, anteroinferior, laterosuperior, lateroinferior, posterosuperior, and posteroinferior lung regions on each side, showed an optimal accuracy [47]. In agreement with this method, scoring (generally from 0 to 3) each area in accordance with the most severe lung ultrasound finding gives a total gravity score (for example, when exploring six regions on each hemithorax a maximum of 36 is reached).

**Figure 4:**
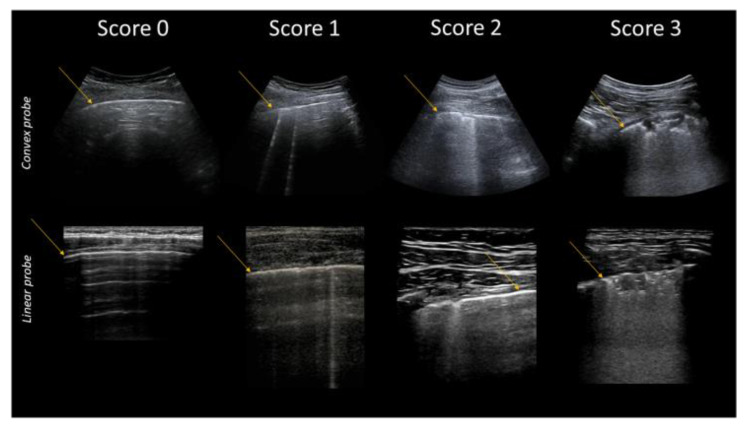
Classification of pathological lung ultrasound findings in COVID-19 patients. Arrows indicate the pleural line. Top: convex probe. Bottom: linear probe.

Clinical and experimental evidence concerning the relationships between pulmonary ultrasound signs and changes in the subpleural histology [5,7,48] allowed the formulation of a specific gravity score of COVID-19, which was initially computed in 14 areas, and published at the beginning of the pandemic [49,50]. This score is synthetized in Table 2 and in Figure 4.

This specific methodology was subsequently validated by two studies attributing a prognostic validity to the US COVID-19 score, and proposing an evidence-based approach through a specific methodology [51,52].

Similarly, using different practical approaches, much literature data supports these original suggestions, showing both the utility of chest ultrasonography in pulmonary COVID-19 and the correlations between the diagnostic CT and ultrasound findings, whether this be at the level of a first diagnosis or as predictive tools in COVID-19 patients.

All in all, LUS represents a valuable tool in symptomatic patients with high negative predictive value for ruling out the disease.

As compared to HRCT, LUS is characterized by a very high sensitivity and specificity in detecting signs of interstitial pneumonia in COVID-19 patients (77–97% and 77–100%, respectively) [53,54,55]. 

From a technical point of view CT and US provide completely different assessments (CT scans lung parenchymal volumes, while in SIS, ultrasound generates a surface density map) and for this reason the diagnostic agreement between LUS and CT in terms of score was not always adequate.

However, in practical terms, LUS can be considered as an equally accurate alternative for CT in many situations where CT is not easily accessible or when molecular tests are not available. The use of lung ultrasound (LUS) as a triage tool has been proposed since the beginning of the COVID-19 pandemic [49] and subsequent studies have confirmed its role [56]. The high sensitivity of ultrasound for the superficial lesions of the lung from the interstitial stages represents its great value. Despite not showing pathognomonic COVID-19-signs, LUS is an established point-of-care tool for the evaluation of patients in the emergency department [57]. Every trained physician evaluating the admitted patients can perform an LUS to make a primary discrimination between subjects with pneumonia and subjects without pneumonia, and to monitor its pulmonary status.

Other aspects are worthy of mention. Asymptomatic carriers represent 17.9–33.3% of patients with COVID-19 [58,59] and they may contribute to the spread of the infection. The yield of screening for COVID-19 with LUS in asymptomatic patients is not known. In a retrospective study 22% of the asymptomatic patients with positive COVID-19 RT-PCR showed LUS findings. In comparison, LUS showed a positive predictive value of 100% [60]. 

The usefulness of LUS to predict complications in COVID-19 pneumonia has been described and sonography seems a powerful predictor of in-hospital mortality, playing a crucial role in risk stratification of patients with COVID-19.

Lung ultrasound score measured at the time of inclusion of the patients was independently associated with admission to the intensive care unit, the need for supplemental oxygen and respiratory support, and mortality. Conversely, a normal scan within 24 h of admission is indicative of a positive evolution of the pathology [61,62,63].

Moreover, LUS involvement in COVID-19 patients correlated with IL-6 levels and with the P/F ratio [64]. In hospitalized COVID-19 patients, pathological LUS was associated with venous thromboembolism [65].

Finally, using the score proposed in [50], a median value higher than 24 was associated with an almost 6-fold increase in the odds of worsening, defined as a combination of high-flow oxygen support, intensive care unit admission, or 30-day mortality as the primary end-point [51].

The worsening of pneumonia in patients with COVID-19 appears in echography with the spatial diffusion of signs of interstitial disease and consolidations. This is in accordance with the proposed grading system. On the contrary, the clinical improvement coincides with the regression of these findings (vertical artifacts, white lung, and consolidations), which leads to a downstaging of the score. Mild signs of interstitial pathology (vertical artifacts) may persist for a long time or indefinitely (see Section 7).

Beyond its use as a diagnostic and prognostic tool, LUS can be used to define optimal PEEP, guide recruitment, or monitor recruitment and should be part of the diagnostic toolset in intensive care units [66]. Ultrasound-guided recruitment is generally carried out according to the principles set out by Bouhemad et al. [67].

US signs of COVID-19 pneumonia are not specific, as they are also present to various degrees in other pathologies. Therefore, the diagnostic accuracy of pulmonary ultrasound in this pathology is strongly influenced by the pretest probability of belonging to an exposed population in a particular epidemiological context, and showing compatible symptoms.

As for any etiology of acute respiratory distress, lung ultrasound must incorporate examinations of the pleura, and of the cardiovascular system, so as to detect myocarditis, for example, and to acquire some hemodynamic data at least.

Five SARS-CoV-2 variants are known—alpha, beta, gamma, delta, and omicron. The delta variant was dominant in the summer of 2021 and the omicron variant was identified in November 2021. There are currently no data demonstrating differences in ultrasound appearance between COVID-19 variants in cases of pneumonia [68].

## 5. Why Does COVID-19 Consolidate the Lung?

Radiographic and ultrasound lung consolidations are characteristic findings in bacterial pneumonia and atelectasis. In viral pneumonia, CT ground-glass opacities (GGO) are usual, while consolidations can be present. These, however, are rare in varicella zoster, Epstein-Barr, paramyxo- and hantavirus pulmonary involvement [37]. Peripheral GGOs are typical in COVID-19, explaining the frequency of SIS in this disease. Consolidations are observed with CT in 72% of COVID-19 patients [43]. Subsegmental consolidative peripheral involvement is present in half of the subjects. Gravitational consolidations, on the other hand, are characteristic of ARDS patients. In ARDS microatelectasis are seen in the early phase. Atelectasis, reduced lung compliance, organizing pneumonia, bacterial infections, and pulmonary fibrosis develop in the fibroproliferative or reparative phases. The distribution of densities results from the generalized increase in weight of the overlying lung causing compression in a sponge like manner [65].

The nature of the lung consolidation in COVID-19 is complex, and theoretically, all the mechanisms described are possible. However, knowledge of the nature of these lesions would have a positive impact on the pharmacological and ventilatory treatment of these subjects.

Gattinoni et al. [69] noted that COVID-19 subjects with respiratory failure showed different patterns of pneumonia with different pathophysiology. At the beginning a “Type L” of pulmonary involvement, characterized by a nearly normal compliance, low pulmonary weight, CT GGO, and low lung recruitability may be seen. The evolution of this phenotype, if it occurs, is towards a “Type H” pattern, showing low compliance, high pulmonary weight, non-aerated tissue, and high lung recruitability.

In COVID-19 the transition between different pulmonary phenotypes probably depends on the interaction of many factors, one of the most important of which could be a maladaptive immune response rather than an increased viral load.

An immune overreaction was described in SARS-CoV-1 and MERS-CoV and in severe influenza (H1N1, H5N1) [70,71]. Cytokine storm causing severe capillary damage and organ dysfunction was supposed. Systemic vasculitis was observed in one report of SARS-CoV-1 [72]. 

Considering the relationships between inflammation, immunity, and coagulation, the evidence of endothelial activation, upregulation of adhesion molecules, endothelial disruption, and activation of coagulation pathways in many bacterial, viral, and parasitic (malaria) diseases is not surprising. In all these situations, an activation of complement component C3 could exacerbate vascular damage. It is interesting to note that excessive complement activation may lead to the activation of a clotting pathway and diffuse thrombotic microangiopathy, and is responsible for a massive local release of pro-inflammatory cytokines [42,73]. 

**Figure 5:**
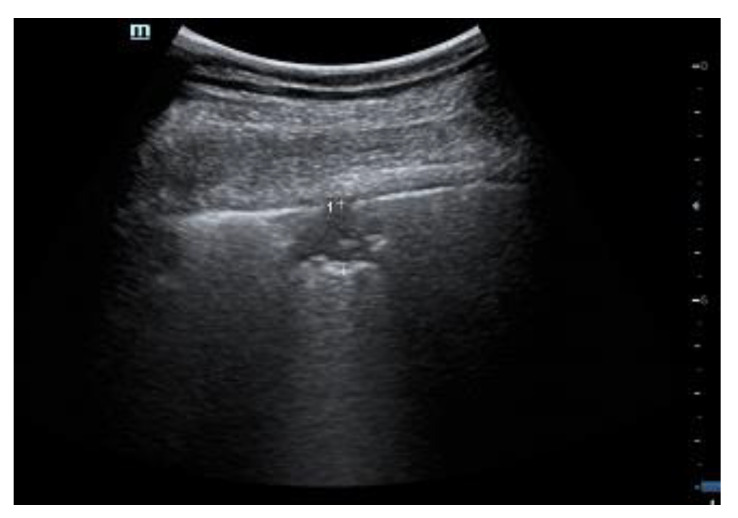
Patient with COVID-19 lung involvement. A small consolidation under the pleura, surrounded by white lung.

In ultrasound, COVID-19 consolidations have the appearance of small cuneiform lesions abutting the pleura, often containing a central echogenic spot of residual air (Score 3), surrounded by white lung (see Figure 5). 

The major consolidations are generally seen in a posterobasal, laterobasal, or superior position, with or without air bronchograms. Observations related to histopathological findings of hemorrhage, vascular fibrin, and cell deposition and clotting have already been discussed. Recently, Tang et al. [74] showed that in 183 patients with confirmed COVID-19, the non-survivors revealed significantly higher D-dimer and fibrin degradation product (FDP) levels compared to the survivors. Therefore, despite atelectasis-organizing pneumonia and complete alveolar exudation maybe being the cause of consolidations, the hypotheses of vascular, thrombotic, or ischemic lung damage which are at the base of some of these findings, should be considered. A published report on three patients with confirmed COVID-19 pneumonia, in which contrast-enhanced ultrasound (CEUS) imaging was conducted to study lung consolidations [75], showed an abnormal early, inhomogeneous, and partial arterial enhancement without evidence of a segmentary arrangement of pulmonary arteries. Consolidations of less than 2 cm did not show enhancement.

These observations suggest the existence of a further pattern of COVID-19 lung involvement in which some consolidations do not represent atelectasis or easily recruitable areas, but rather tissue with large perfusion defects. Further confirmations of these observations would imply a complete review of the therapies for COVID-19, which should be finely tuned not only as regards the antiviral approach, but also regarding the selective ventilation methods and valid treatments to interrupt the many targets of the maladaptive immune response.

In this context, ultrasound (B-mode and CEUS) can play a key role as a predictive instrument of aggravation by detecting and characterizing the consolidations, and as a tool for managing a targeted therapy and ventilation.

## 6. COVID-19 in Pediatrics

During the COVID-19 pandemic, LUS played an important role in screening affected individuals and also, to a lesser extent, the pediatric population [76]. The clinical COVID-19 manifestations in children are mild or moderate compared to adults. Dong et al. [77] reported that approximately 4% of children were asymptomatic, 51% had a mild illness, 39% had a moderate illness, and 6% had a severe or critical illness. In those pediatric patients who contracted COVID-19, vertical artifacts, pleural irregularities, subpleural consolidations, and patchy white lung were described. Musolino et al. [78] confirmed the presence of bilateral lung involvement in 70% of the patients and pleural irregularities in 60% of the patients. Children with a moderate disease presented more vertical artifacts than patients with a mild disease (85.7% vs. 36.4%, respectively). No pleural effusion was detected. Denina et al. [79] noted subpleural consolidations in 25% of cases and confluent B-lines in 62%. The existing studies confirm that LUS findings in children are similar to those described in adults and are not specific for the COVID-19 disease.

## 7. Post-Acute Sequelae of COVID-19 Pneumonia

Little is known about the possible clinical complications persisting after the resolution of acute COVID-19. Among hospitalized patients with COVID-19 respiratory impairment, over 50% show abnormalities on CT and the latter are more common in subjects with a more severe pulmonary disease [80].

The most common abnormalities are ground-glass opacity, densities in the form of subpleural bands, reticular thickening, and evidence of fibrotic changes with air trapping. It has been speculated that some patients could progress to advanced lung fibrosis or post-COVID interstitial lung disease [81].

In view of the high sensitivity of pulmonary ultrasound in detecting parenchymal alterations in primary and secondary fibrosing diseases of the lung [3], it is conceivable that LUS may have a role in post-COVID patient surveillance. In some studies LUS showed an outstanding discrimination ability compared to CT in identifying fibrotic changes in the post-COVID-19 follow-up [82]. LUS should be proposed as the first-line tool in follow-up programs, while chest CT could be performed based on LUS findings.

## 8. Conclusions

Lung ultrasound is nowadays a rapidly evolving field. The COVID-19 pandemic has contributed to a better understanding of its technical basis and its clinical use. In this review we highlighted known clinical applications, as well as US findings and applications of lung US in the context of the current COVID-19 pandemic.

Chest US is used to a great extent in the bedside management of COVID-19 pneumonia (home, emergency department, ward, and ICU) and for monitoring the evolution of lung lesions. Lung US is comparable to CT for detecting the superficial parenchymal involvement in COVID-19 and its severity. Moreover, based on the ultrasound findings, a prognostic stratification of the patients can be implemented.

In the face of a general poor specificity of ultrasound signs in COVID-19 pneumonia, there is a need for a better understanding of the physical mechanisms that determine the image formation starting from the received ultrasound signals, together with the use of a dedicated methodology and signal-processing procedures.

## Figures and Tables

**Table 1:** Differences between acute cardiogenic pulmonary edema (ACPE) and ARDS (pneumogenic) sonographic interstitial syndrome (SIS).

Cardiogenic	Pneumogenic
Diffuse homogenous SIS	Diffuse inhomogeneous SIS, and spared areas
Smooth, linear, and regular pleural line	Coarse, irregular, and cobbled pleural line
Bright (laser-like), and modulated artifacts	Rough attenuated vertical artifacts
Normal sliding sign	Reduced sliding sign

**Table 2:** COVID-19 Scores.

Score	Description
0	Pleural line is regular. Horizontal artifacts and mirror effects are present. Normal lung.
1	Pleural line has slight alterations with sporadic vertical bright artifacts. The presence of relatively small acoustic channels due to focal interstitial thickening is speculated.
2	Pleural line has relevant alterations. Progression of subversion of peripheral air space geometry causes a predominance of vertical artifacts. Small subpleural consolidations, related to deaeration, can be present.
3	Pleural line is irregular and cobbled. Subpleural lung is denser and more disordered. White lung with or without larger consolidations may be present. Small and large consolidations are subpleural regions minimally or completely deprived of air.
